# Identification and Validation of a Hypoxia and Glycolysis Prognostic Signatures in Lung Adenocarcinoma

**DOI:** 10.7150/jca.91504

**Published:** 2024-01-21

**Authors:** Wenhao Zhao, Chen Ding, Meiru Zhao, Yongwen Li, Hua Huang, Xuanguang Li, Qian Cheng, Zijian Shi, Weining Gao, Hongyu Liu, Jun Chen

**Affiliations:** 1 Department of Lung Cancer Surgery, Tianjin Medical University General Hospital, Tianjin, China.; 2 Department of Dermatovenereology, Tianjin Medical University General Hospital/ Tianjin Institute of Sexually Transmitted Disease, Tianjin, China.; 3 Tianjin Key Laboratory of Lung Cancer Metastasis and Tumor Microenvironment, Tianjin Lung Cancer Institute, Tianjin Medical University General Hospital, Tianjin, China.

**Keywords:** lung adenocarcinoma, hypoxia, glycolysis, immune, prognosis, immunotherapy.

## Abstract

**Background:** Lung adenocarcinoma (LUAD) represents a prevalent subtype of non-small cell lung cancer with a complex molecular landscape. Dysregulated cellular energetics, notably the interplay between hypoxia and glycolysis, has emerged as a hallmark feature of LUAD tumorigenesis and progression. In this study, we aimed to identify hypoxia and glycolysis related gene signatures and construct a prognostic model to enhance the clinical management of LUAD.

**Methods:** A gene signature associated with hypoxia and glycolysis was established within the The Cancer Genome Atlas (TCGA) cohort and subsequently validated in the GSE31210 cohort. Additionally, a nomogram was formulated to aid in predictive modeling. Subsequently, an evaluation of the tumor microenvironment and immune checkpoints expression levels was conducted to discern disparities between low risk and high risk groups. Lastly, an exploration for drugs with potential effectiveness was carried out.

**Results:** Our analyses revealed a distinct hypoxia and glycolysis related gene signature consisting of 6 genes significantly associated with LUAD patient survival. Integration of these genes into the prognostic model demonstrated superior predictive accuracy for patient outcomes. Furthermore, we developed a user-friendly nomogram that effectively translates the model's prognostic information into a practical tool for clinical decision-making.

**Conclusion:** This study elucidates the critical role of hypoxia and glycolysis related genes in LUAD and offers a novel prognostic model with promising clinical utility. This model has the potential to refine risk stratification and guide personalized therapeutic interventions, ultimately improving the prognosis of LUAD patients.

## Introduction

Lung adenocarcinoma (LUAD), a histological subtype of non-small cell lung cancer, continues to pose a significant global health challenge, characterized by its high incidence and frequently unfavorable prognosis 1. In recent years, significant strides have been made in understanding the molecular underpinnings of LUAD, revealing that dysregulated cellular energetics as a key player in tumor initiation and progression. Among the pivotal aspects of cellular energetics, the intricate interplay between hypoxia and glycolysis has emerged as a central focus of research in the context of LUAD.

Hypoxia, characterized by inadequate oxygen supply to tissues, is a frequent occurrence in solid tumors, including LUAD, owing to the chaotic and disorganized nature of tumor vasculature 2, 3. It has long been recognized as a potent driver of tumor aggressiveness, cell apoptosis, promoting metastasis, immune evasion, and resistance to therapy 4, 5. In response to hypoxic conditions, cancer cells undergo a series of adaptive changes to ensure their survival and proliferation. These changes include the stabilization of hypoxia-inducible factors (HIFs), which orchestrate a transcriptional program that enhances glycolysis, angiogenesis, and other pro-tumorigenic processes 6-8. Consequently, the reliance on glycolysis for energy production, even in the presence of oxygen (a phenomenon known as the Warburg effect), becomes a hallmark feature of hypoxic tumor microenvironments (TME) 9.

Glycolysis, the anaerobic metabolic pathway that converts glucose into pyruvate, represents an integral facet of cancer cell metabolism 10. Beyond its role in energy generation, glycolysis provides cancer cells with intermediates necessary for biomass production and supports redox homeostasis. The glycolytic reprogramming in cancer cells serves a dual purpose: it meets bioenergetic requirements while also playing a pivotal role in the creation of an immunosuppressive microenvironment, rendering tumor cells less susceptible to immune surveillance 11-13. Thus, the intricate crosstalk between hypoxia and glycolysis in LUAD creates a fertile ground for tumorigenesis, progression, and resistance to therapy.

As a testament to the clinical significance of hypoxia and glycolysis in LUAD, numerous studies have investigated their effects on patient outcomes 14, 15. However, the precise molecular mechanisms governing their interplay and their collective impact on LUAD prognosis remain incompletely understood. In this context, there is a pressing need for a comprehensive assessment of hypoxia and glycolysis related gene signatures that can refine prognostic stratification and guide clinical decision-making for LUAD patients. The advent of high-throughput transcriptomic technologies and the availability of genomic datasets, such as The Cancer Genome Atlas (TCGA), have revolutionized our ability to explore the molecular intricacies of cancer. Leveraging these resources, we have embarked on a comprehensive analysis aimed at identifying hypoxia and glycolysis related gene signatures in LUAD. By integrating multi-omics data and employing sophisticated bioinformatic methodologies, we seek to unravel the molecular underpinnings of LUAD tumorigenesis driven by dysregulated cellular energetics.

In this study, we present our findings regarding the identification of a distinct hypoxia and glycolysis related gene signature in LUAD and the subsequent construction of a prognostic model. This model is poised to offer a refined understanding of LUAD patient outcomes, allowing for more personalized and effective therapeutic strategies. Furthermore, we have developed a user-friendly nomogram based on this model, facilitating its clinical application for risk assessment and patient management.

## Materials and Methods

### Data Acquisition

The data pertaining to LUAD in this study were sourced from the TCGA and the Gene Expression Omnibus (GEO). The TCGA-LUAD cohort encompassed a total of 489 patient records, comprising both profiling data and corresponding clinical information, which were retrieved from the TCGA official website (https://cancergenome.nih.gov). Additionally, gene expression profiles of 59 normal samples, representing adjacent non-tumor tissue from the same patients, were obtained from TCGA. The expression matrix and clinical data from the GSE31210 dataset were downloaded from GEO, and this dataset was built upon the GPL570 platform, including 226 LUAD samples. Patients meeting the following selection criteria were included: (a) histologically diagnosed with LUAD, (b) having available gene expression data, and (c) possessing accessible survival and clinical information. Patients sourced from TCGA-LUAD comprised the training cohort, while those from GSE31210 served as an external validation cohort.

### Identification of Hypoxia Status

T-distributed Stochastic Neighbor Embedding (t-SNE) and K-Means Clustering (K-means) algorithms were employed to determine the hypoxia status of tumor samples, accessible through the “Rtsne” and “k-means” R package. T-SNE, a nonparametric and unsupervised method, which divides or condenses patients into distinct clusters based on provided signatures or hallmarks. This study incorporated 59 genes as established hallmarks of hypoxia, derived through a two-step procedure. Initially, 200 genes were collected from hypoxia-related hallmark gene sets within the Molecular Signatures Database (MsigDB V7.4). Supplementary Table S1 contains the supplemented list. Subsequently, a selection process was carried out using univariate Cox regression analysis, executed through the “Survival” R package, resulting in the identification of the hypoxia related genes employed in this investigation. Utilizing the aforementioned algorithms, patients were categorized into distinct groups based on their hypoxia status. Two groups (namely, group 1 and group 2) were chosen for the evaluation of hypoxia status. Kaplan-Meier plots were generated for the two groups to compare their overall survival (OS).

### Identification of Glycolysis Status

295 GRGs were identified from the “HALLMARK GLYCOLYSIS” “REACTOME GLYCOLYSIS” “WP GLYCOLYSIS AND GLUCONEOGENESIS” “KEGG GLYCOLYSIS GLUCONEOGENESIS” gene sets in the MsigDB database. Supplementary Table S2 contains the supplemented list. We utilized the “ConsensusClusterPlus” 16 R package, employing the k-means machine learning algorithm, to conduct an unsupervised consensus clustering. This approach enables the segmentation or consolidation of cases into multiple distinct clusters, according to the provided hallmarks or signatures. Besides, hallmark gene sets summarize and represent specific well-defined biological states or processes and display coherent expression. In detail, we used the consensus clustering algorithm with 1,000 iterations by sampling 80% of the data in each iteration. The ideal number of clusters was determined through a comprehensive assessment, considering the Item-Consensus plot, the Proportion of Ambiguous Clustering (PAC) algorithm, and the relative change in the area under the cumulative distribution function (CDF) curves. Two clusters (namely, cluster 1 and cluster 2) were selected for assessing glycolysis status. Kaplan-Meier plots were generated for the two clusters to compare their OS.

### Differentially Expressed Genes (DEGs)

The “limma” 17 R package was employed to identify preliminary hypoxia and glycolysis related DEGs. Similarly, DEGs were identified between tumor samples and normal samples. Next, intersect the hypoxia and glycolysis related DEGs with tumor and normal related DEGs for subsequent analysis.

### Functional Enrichment Analysis

To comprehend the functions and pathways of the aforementioned DEGs as detailed above, KEGG pathway analysis and GO enrichment analysis were performed using the “clusterProfiler” 18 R package. Statistical significance was determined by considering p-values and FDR values both below 0.05. Additionally, gene set enrichment analysis (GSEA) was employed to identify notably enriched pathways in both groups, utilizing the gene set “c2.cp.kegg.v7.4.symbols.gmt”.

### Construction and Validation of a Hypoxia and Glycolysis Related Prediction Model

A hypoxia and glycolysis related prediction model was established via univariate Cox and least absolute shrinkage and selection operator (LASSO) regression, by using the “Survival” and “Glmnet” 19 R packages. The pivotal genes and their respective coefficients were derived from these analyses to facilitate model construction. Subsequently, the risk score for each patient was computed by utilizing the standardized expression levels of these pivotal genes and their associated regression coefficients. The risk score was determined using the formula: risk score = 0.000126 × (expression of GAPDH) + 0.000599 × (expression of FSCN1) + 0.002546 × (expression of SLC2A1) + 0.002353 × (expression of FAM83A) + 0.001549 × (expression of PLEK2) + 0.009292 × (expression of GJB3). To evaluate survival disparities between high risk and low risk groups, Kaplan-Meier survival analysis was conducted employing the “survival” R package.

### Establishment of a Nomogram Scoring System

A nomogram was constructed using the “rms” R package, including the risk score computed previously, and some clinical characteristics, such as pathological grade, clinical stage, age and gender. To assess the predictive efficacy of the model, we compared it to the performance of these clinical features. Time-dependent Receiver Operating Characteristic (ROC) curves were generated using the “survivalROC” 20 R package. And calibration plots were generated to evaluate the nomogram's calibration and discrimination properties by utilizing the “rms” R package. Furthermore, we also applied ROC analysis and calibration plots to the external validation dataset GSE31210.

### Estimation of TME

The immune cell expression and 13 immune-related pathways in LUAD were quantified using the ESTIMATE and single-sample GSEA (ssGSEA) analysis. The comparison of checkpoint expression levels between the high risk and low risk groups was carried out through the utilization of the “ggpubr” R package.

### Tumor Mutational Burden (TMB) and Drug Sensitivity Analysis

TMB serves as an emerging therapeutic indicator for assessing the sensitivity of immunotherapy, and defined as the frequency of specific mutations within a tumor's genetic makeup, was determined for each LUAD case as previously described 21. To assess the association between the risk score and TMB, the Wilcoxon rank-sum test was employed. Subsequently, the “maftools” 22 package facilitated the identification of the top 20 mutated genes and enabled the visualization of mutations and their frequencies across all samples in the TCGA cohort. Tumor Immune Dysfunction and Exclusion (TIDE) stands as a computational framework that integrates T cell dysfunction expression signatures and T cell exclusion to model tumor immune evasion. This model allows for the prediction of immunotherapy outcomes through two distinct approaches 23-25. Importantly, we conducted an examination to ascertain the correlation of our signature with TIDE. The drug distribution within the high and low risk score groups was subject to analysis and visualization through the “pRRophetic” 26 R package. And the Wilcoxon test was used to compare the difference of IC50 between the high risk and low risk groups.

## Results

### Identification of Hypoxia Status and Glycolysis Status

**Figure 1:**
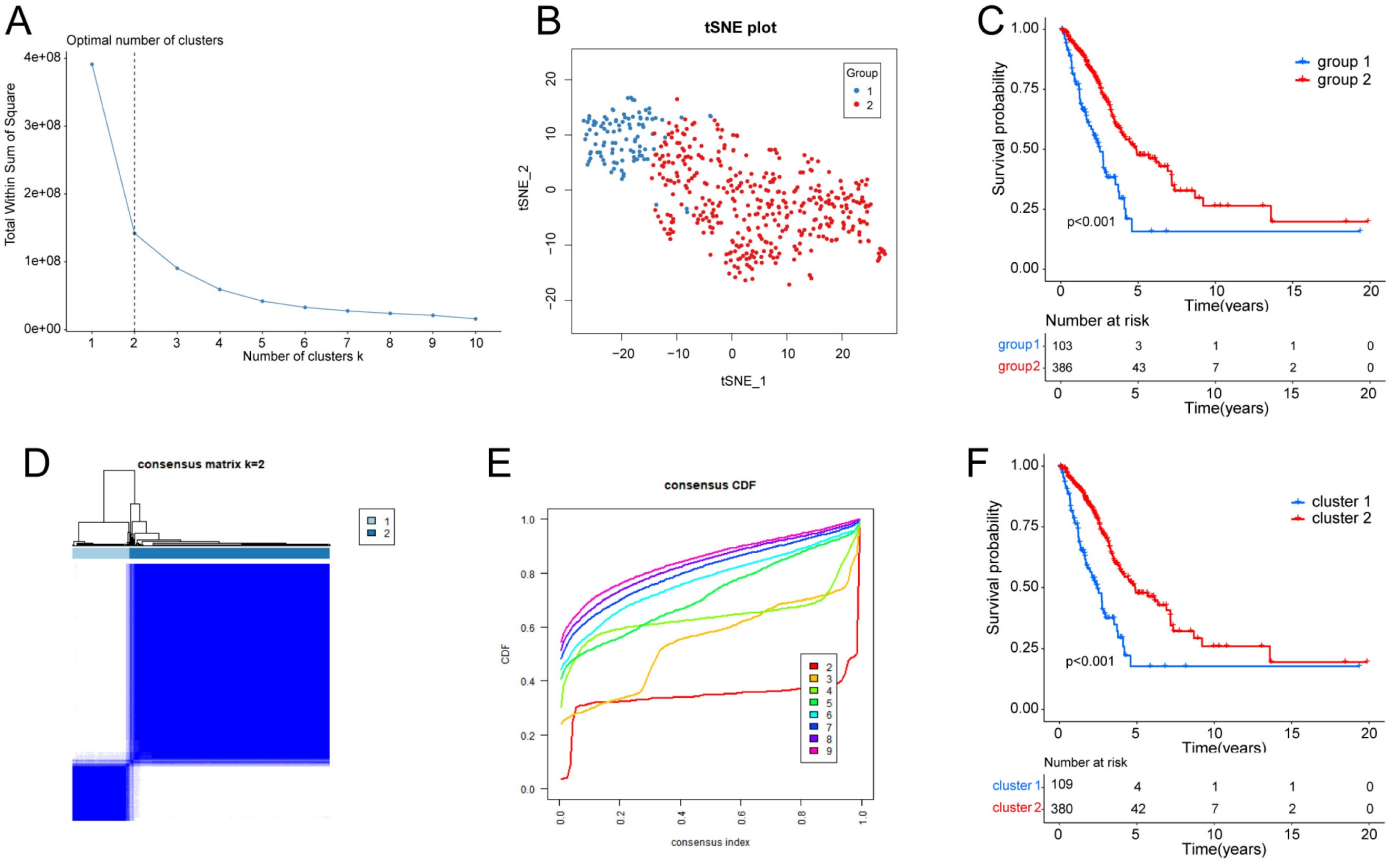
** Identification of hypoxia status and glycolysis status. (A)** The total Within Sum of Square (WSS) when k = 1-10. **(B)** Dot plot for two distinct clusters identified by t-SNE and K-means algorithms based on 200 hypoxia-related genes. **(C)** Survival analysis (Kaplan-Meier) of overall survival (OS) for patients in two hypoxia related clusters. **(D)** Consensus matrix heatmap defining two glycolysis related clusters (k = 2) and their correlation area. **(E)** Cumulative distribution function (CDF) when k = 2-9. (F) Survival analysis (Kaplan-Meier) of OS for patients in two glycolysis related clusters.

Supplementary Fig. S1 depicts the workflow diagram. To elucidate the detailed mechanisms in cancer patients with different hypoxia status, the TCGA samples were divided into two groups based on their hypoxia status using the t-SNE algorithm, resulting in 103 samples in group 1 and 386 samples in group 2 (Fig. 1A, B). Subsequent Kaplan-Meier survival analysis revealed that the OS of group 2 was better (Fig. 1C). As for the LUAD subtypes associated with glycolysis related genes, we conducted consensus clustering analysis on the expression profiles of glycolysis related genes. And “K = 2” was the optimal number of clusters, in this situation, the difference between groups was the smallest, and the difference outside the group was the largest. Accordingly, we accurately classified LUAD patients into two subtypes, denoted as cluster 1 and cluster 2 (Fig. 1D). This partitioning yielded a stable distribution, as evident from the relative change in the area under the CDF curve (Fig. 1E). Subsequent Kaplan-Meier survival analysis revealed that the OS of cluster 2 was better (Fig. 1F).

### DEGs Based on Hypoxia and Glycolysis Status

**Figure 2:**
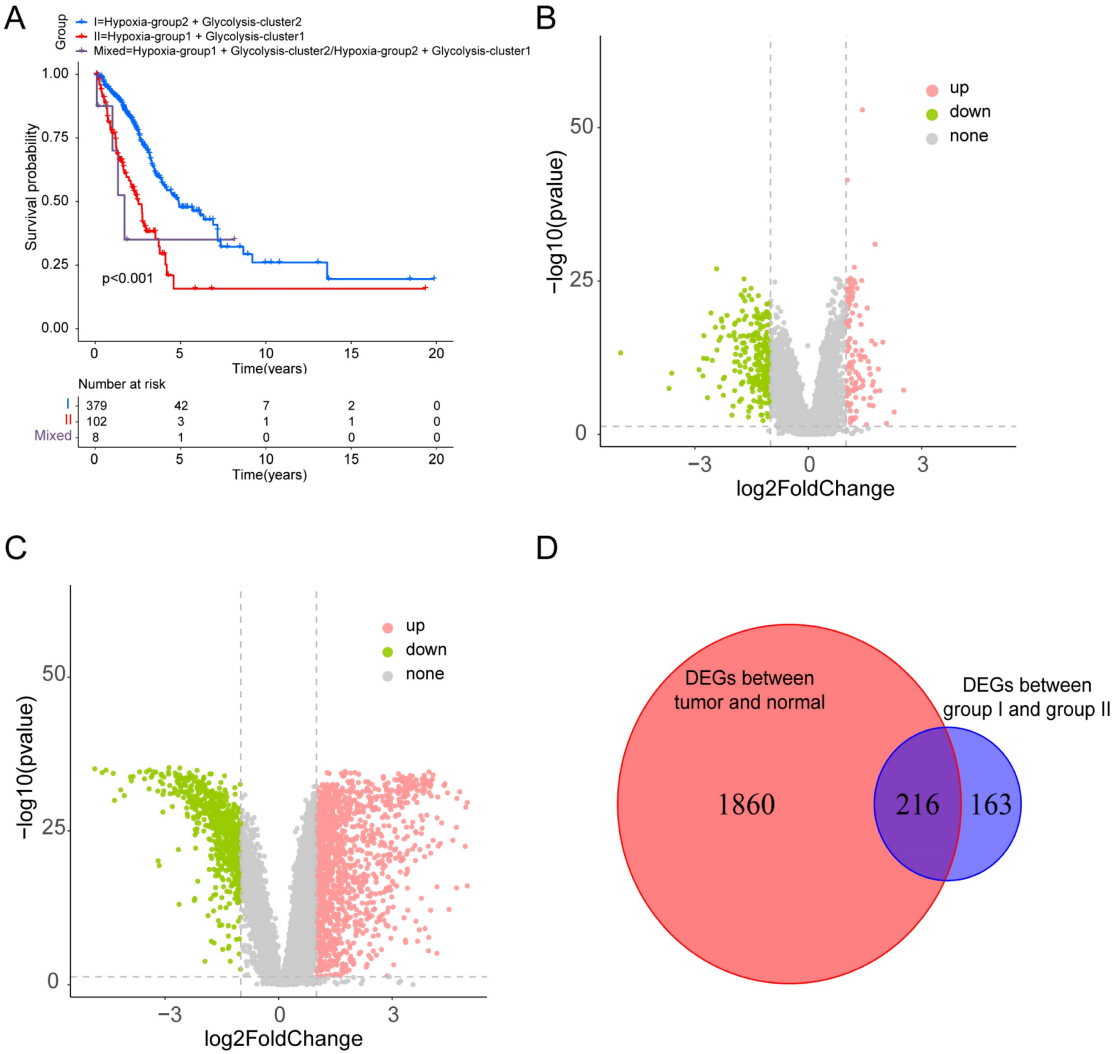
** Dividing into groups and getting differentially expressed genes (DEGs) based on hypoxia and glycolysis status. (A)** Kaplan-Meier plot of OS for patients in group I, group II, and group Mixed. **(B)** Volcano plot showing the DEGs between group I and group II. **(C)** Volcano plot showing the DEGs between tumor samples and normal samples. **(D)** Venn diagrams showing overlaps of hypoxia-glycolysis related DEGs and tumor-normal related DEGs.

Based on the identified hypoxia and glycolysis status, we integrated them into a two-dimensional index, which allowed for the classification of TCGA patients into three distinct groups: (1) group I was the patients with better prognosis in both hypoxia status and glycolysis status (2) group II was the patients with worse prognosis in both hypoxia status and glycolysis status and (3) group Mixed was the remaining patients. The Survival analysis revealed a noteworthy disparity among the three groups, with patients in group I had the best prognosis, whereas patients in group II had the worst prognosis (Fig. 2A).

To identify the DEGs associated with hypoxia and glycolysis status, we first overlapped the TCGA and GSE31210 database, and obtain 18341 genes for subsequent analysis. Then we performed DEGs between the two groups. These analyses initially yielded 379 DEGs (Fig. 2B). To narrow down the scope of the DEGs, we conducted a comparison between tumor samples and normal samples (Fig. 2C). Then we overlapped the hypoxia-glycolysis related DEGs and tumor-normal related DEGs. This process culminated in the identification of 216 hypoxia and glycolysis related DEGs (Fig. 2D).

### Pathway Enrichment Analysis

**Figure 3:**
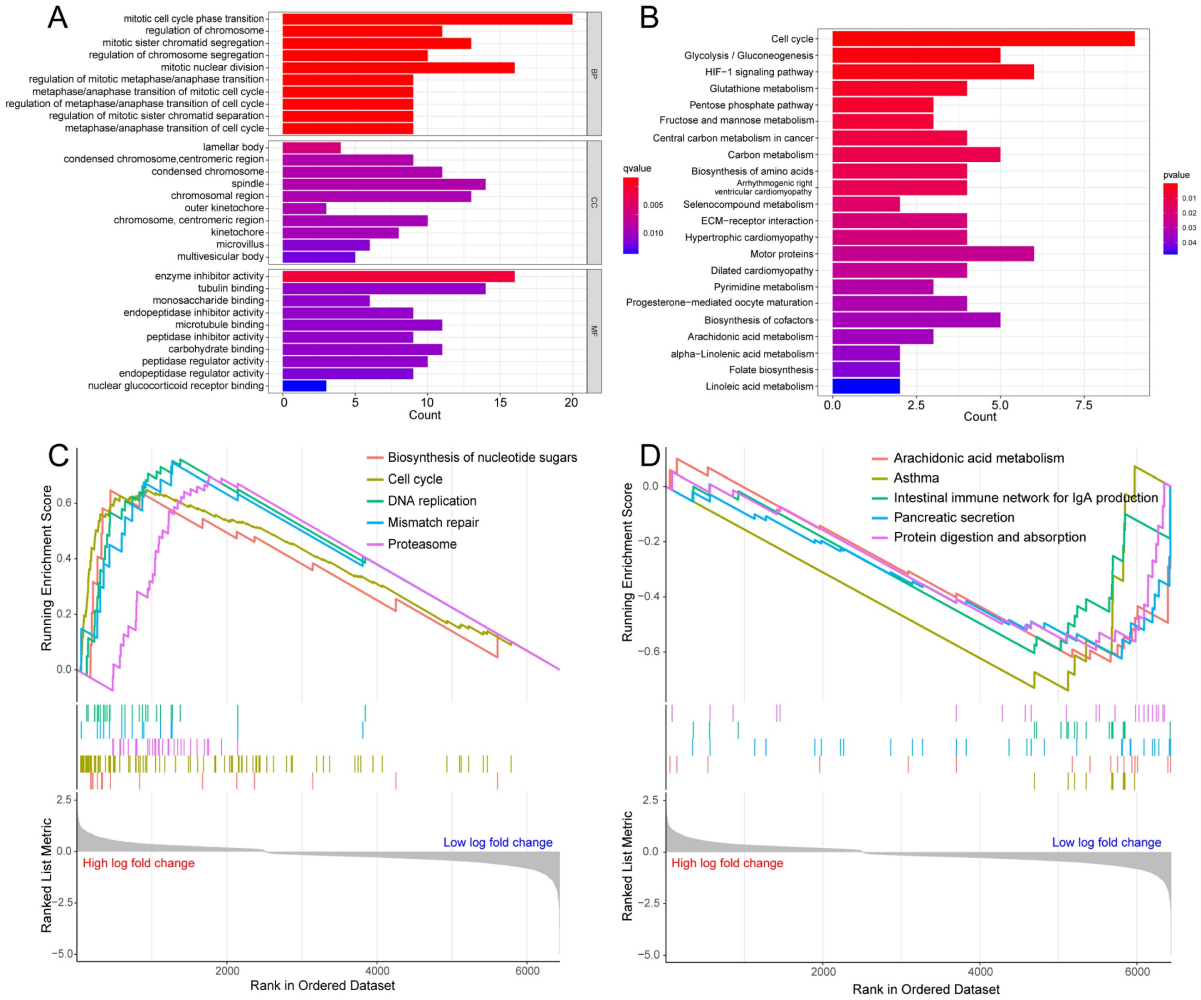
** Enrichment analysis for the hypoxia and glycolysis related DEGs. (A)** Column diagrams of Gene Ontology analysis for hypoxia and glycolysis related DEGs. **(B)** Column diagrams of Kyoto Encyclopedia of Genes and Genomes analysis for hypoxia and glycolysis related DEGs. **(C, D)** The enriched gene terms in gene set enrichment analysis.

To probe the potential biological behavior of the hypoxia and glycolysis related subtypes, we proceeded GO function enrichment analysis and KEGG pathway enrichment analysis. The GO enrichment analysis revealed that pathways were highly enriched on mitotic cell cycle phase transition, mitotic nuclear division, mitotic sister chromatid segregation, regulation of chromosome, and the regulation of chromosome segregation (Fig. 3A). KEGG analysis revealed that DEGs were mostly associated with Cell cycle, Glycolysis/Gluconeogenesis, HIF-1 signaling pathway, Glutathione metabolism, and the Pentose phosphate pathway (Fig. 3B). GSEA showed that the up-regulated genes were significantly enriched on Biosynthesis of nucleotide sugars, Cell cycle, DNA replication, Mismatch repair, and the Proteasome. And the down-regulated genes were significantly enriched on Arachidonic acid metabolism, Asthma, Intestinal immune network for IgA production, Pancreatic secretion, and Protein digestion and absorption (Fig. 3C, D).

### Construction and Validation of the Hypoxia and Glycolysis Related Prognostic Model

**Figure 4:**
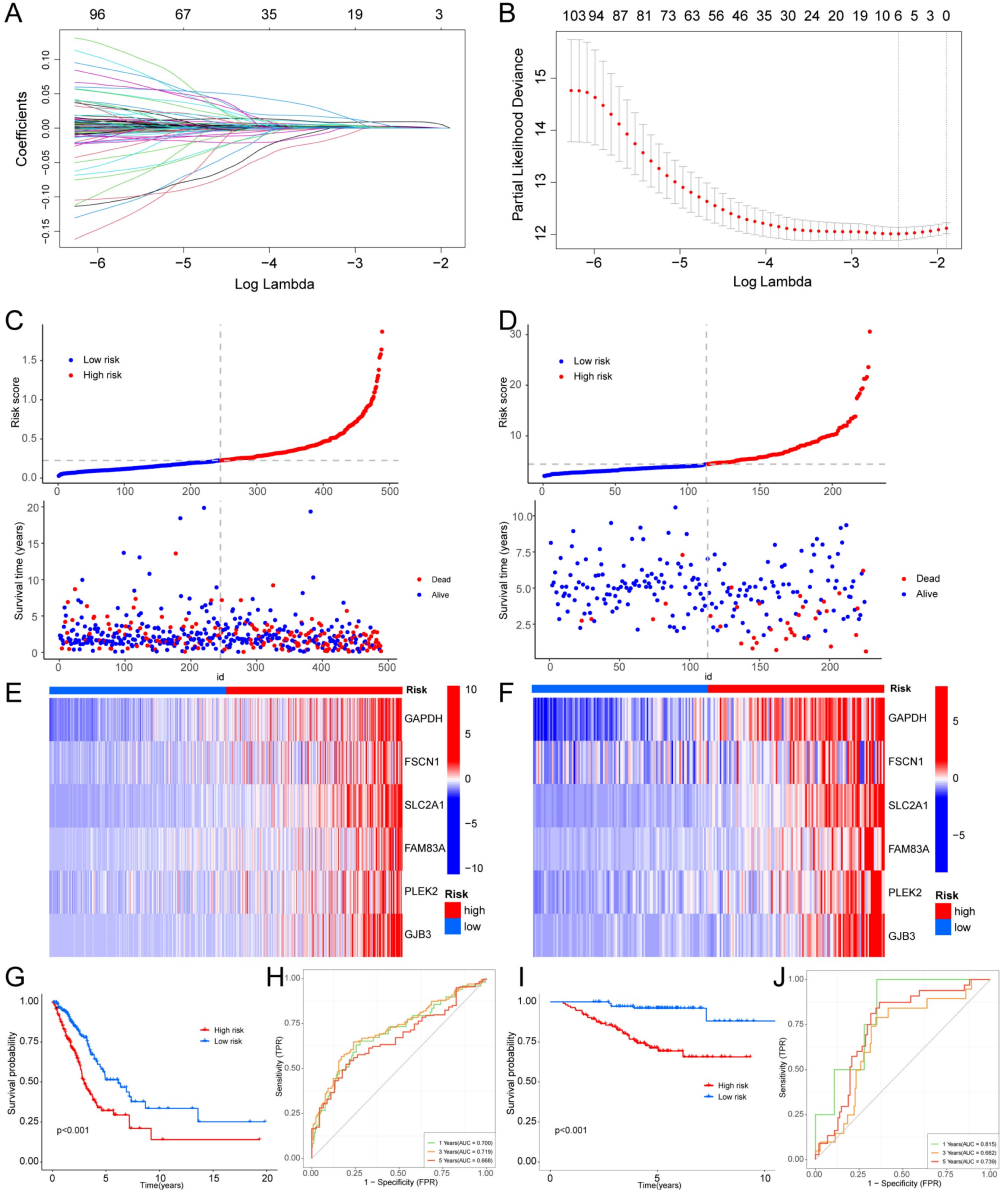
** Construction of a hypoxia and glycolysis related prognostic model. (A, B)** Determination of the number of factors by the LASSO analysis. **(C, D)** The risk score and survival status distribution diagrams of the high risk and low risk groups in the TCGA and GSE31210. **(E, F)** Heat map of the expression profiles of members in the selected 6 genes. **(G, I)** Kaplan-Meier plots of OS for patients in the high risk and low risk groups. **(H, J)** The area under the curve (AUC) of the ROC curve shows the accuracy of the predictive survival signature.

We employed univariate Cox regression and LASSO regression to identify a set of six signature genes (GAPDH, FSCN1, SLC2A1, FAM83A, PLEK2, and GJB3) from the hypoxia and glycolysis related genes to construct a prognostic model (Fig. 4A, B). Supplementary Table S3 and S4 contains the supplemented list. The expression levels of GAPDH, FSCN1, SLC2A1, FAM83A, PLEK2, and GJB3 all presented significant differences between tumor tissues and normal tissues (Supplementary Fig. S2). Patients were divided into high risk and low risk groups based on the median risk score (0.2256). And the patients in the validation cohort GSE31210 were also divided into high risk and low risk groups by the same way. Risk score and the distributions of survival status are shown in Fig. 4C, D. The heatmap showed that GAPDH, FSCN1, SLC2A1, FAM83A, PLEK2, and GJB3 were upregulated in the high risk group (Fig. 4E, F). The OS between patients from these two groups has a significant difference, which meant that patients with a high risk score had a higher mortality rate (Fig. 4G, I). The ROC curves of the TCGA cohort showed that the area under the curve was 0.700, 0.719, and 0.668, and the validation cohort was 0.815, 0.682, and 0.739, for 1-, 3-, and 5-years OS rates, respectively (Fig. 4H, J). The PCA analyses showed good results of this prognosis model (Supplementary Fig. S3).

We conducted a clinical subgroup analysis encompassing conventional clinicopathological characteristics, such as age (>65 and ≤65 years), sex (Female and Male), clinical stage (I-II and III-IV), T stage (T1-2 and T3-4), M stage (M0 and M1), and N stage (N0 and N1-3). This analysis revealed the robust accuracy of the signature in predicting the prognosis for nearly all LUAD patients (Supplementary Fig. S4, 5). A heatmap was created to further understand the differences in clinical characteristics and the hypoxia and glycolysis related genes expression between high risk and low risk groups, which revealed the number of patients with high T stage, N stage and clinic stage in the high risk group is higher, and male patients were more common in high risk group (Supplementary Fig. S6). The distribution of LUAD patients with different groups according to each clinical feature in the TCGA cohort is shown in Table 1. Association studies revealed a significant correlation between clinical stage and different risk groups in the GSE31210 cohort, as well as a significant correlation between gender and different risk groups (Table 2).

### Construction of a Prognostic Nomogram

**Figure 5:**
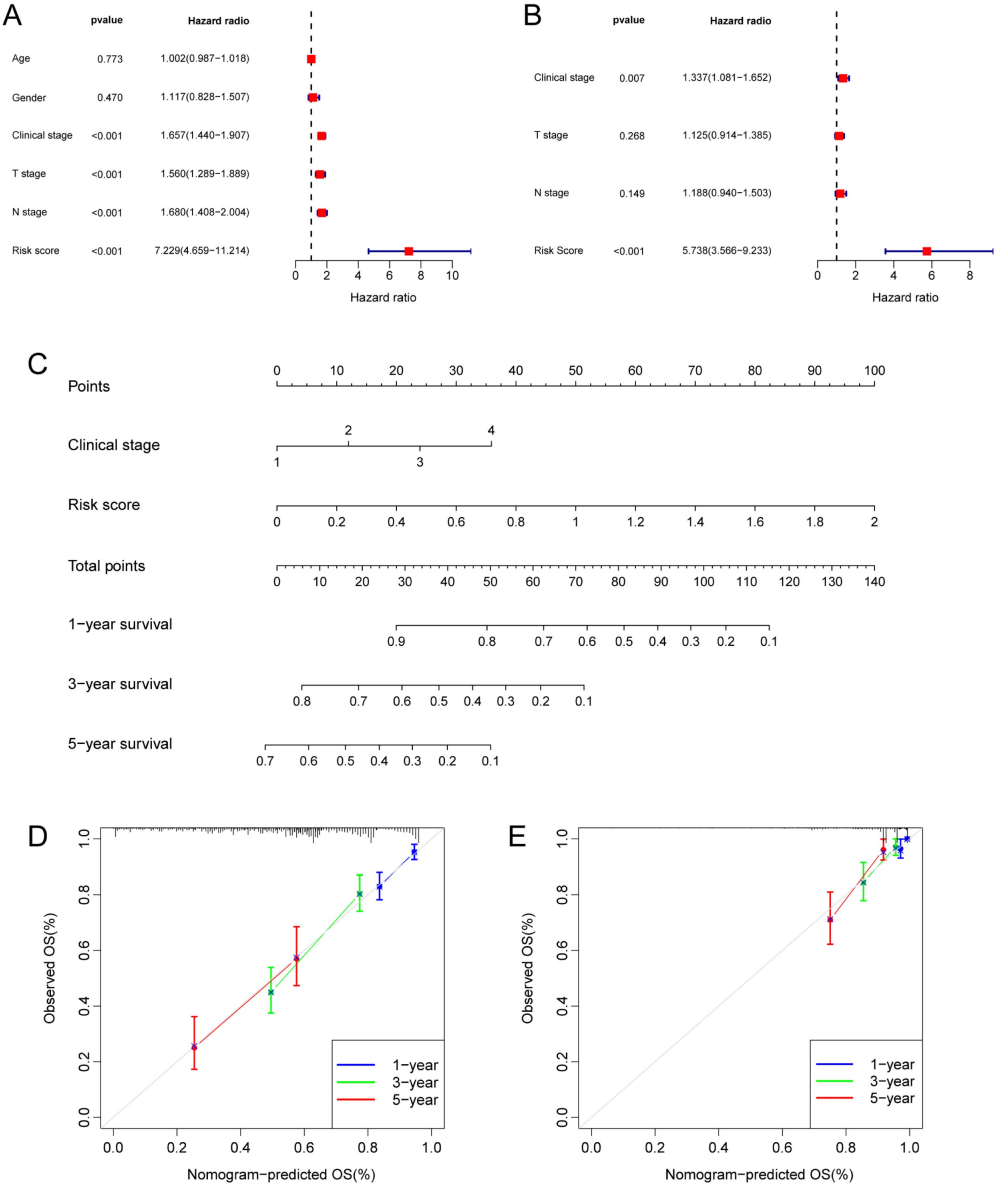
** Independent prognostic analysis of the hypoxia and glycolysis related prognostic model and construction of nomogram and calibration. (A)** The hazard ratio (HR) and 95% confidence interval of risk score and all clinical features were calculated using the univariate Cox regression analysis. **(B)** The HR and 95% confidence interval of risk score and all clinical features were calculated using the multivariate Cox regression analysis. **(C)** The nomogram that includes the risk score and clinical stages predicted the probability of the 1-, 3-, and 5-years OS. **(D, E)** The calibration curves for the predicted 1-, 3-, and 5-years OS rates in TCGA cohort and GSE31210 cohort.

Taking into account the clinicopathological characteristics, we conducted both univariate and multivariate Cox regression analyses. These analyses identified risk score and clinic stage as independent factors significantly impacting the prognosis of LUAD patients (Fig. 5A, B). Furthermore, we developed a nomogram and conducted calibration (Fig. 5C), demonstrating that it could reasonably predict the 1-, 3-, and 5-year OS rates when compared to an ideal model in both the TCGA and GSE31210 datasets (Fig. 5D, E).

### TME and Immune Checkpoint Analysis

**Figure 6:**
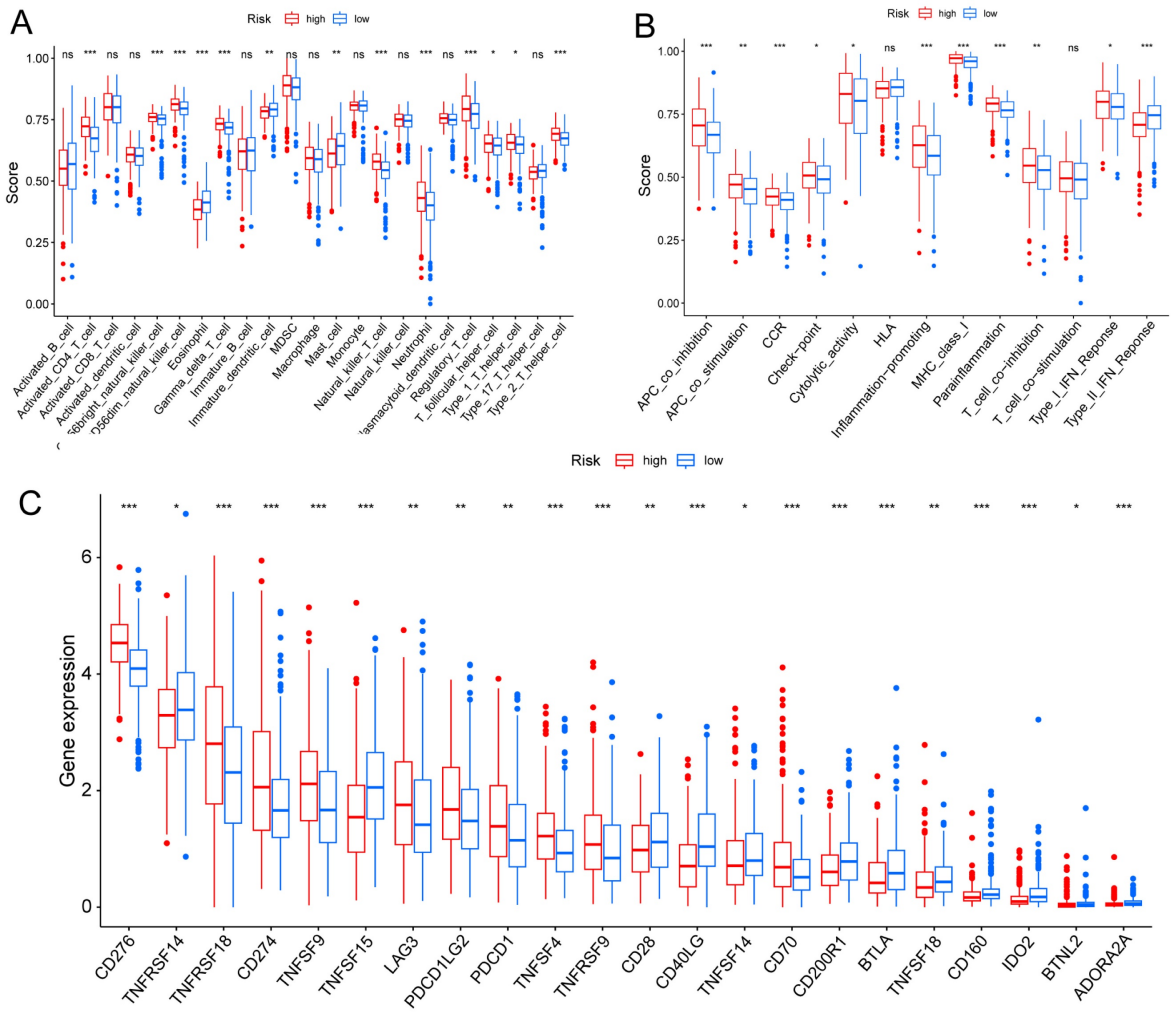
** TME, and checkpoint analysis in LUAD. (A)** The box plots of immune cells between the high risk and low risk groups. **(B)** The box plots of immune related pathways between the high risk and low risk groups. **(C)** The box plots of checkpoint related genes between the high risk and low risk groups. ^ns^p ≥ 0.05, *p < 0.05, **p < 0.01, ***p < 0.001.

TME is crucial for the occurrence and development of tumors. We assessed the expression levels of infiltrating immune cells and pathways and found that the expression of Activated-CD4-T-cell, CD56bright-natural-killer-cell, CD56dim-natural-killer-cell, Gamma-delta-T-cell, Natural-killer-T-cell, Neutrophil, Regulatory-T-cell, T-follicular-helper-cell, Type-1-T-helper-cell, and Type-2-T-helper-cell were higher in the high risk group (Fig. 6A). Moreover, the expression of APC-co-inhibition, APC-co-stimulation, CC chemokine receptor (CCR), check-point, Cytolytic-activity, Inflammation-promoting, MHC-class-I, parainflammation, T-cell co-inhibition, and Type-I-IFN-Response was higher in the high risk group (Fig. 6B). Thus, the levels of checkpoint genes between the high risk and low risk groups were evaluated, which revealed that about half of the genes were highly expressed in high risk group, such as CD276, TNFRSF18, CD274, TNFSF9, TNFSF4, TNFRSF9 (Fig. 6C).

### The Role of Risk Score Participating in Immunotherapy and Drug Sensitivity Analysis

**Figure 7:**
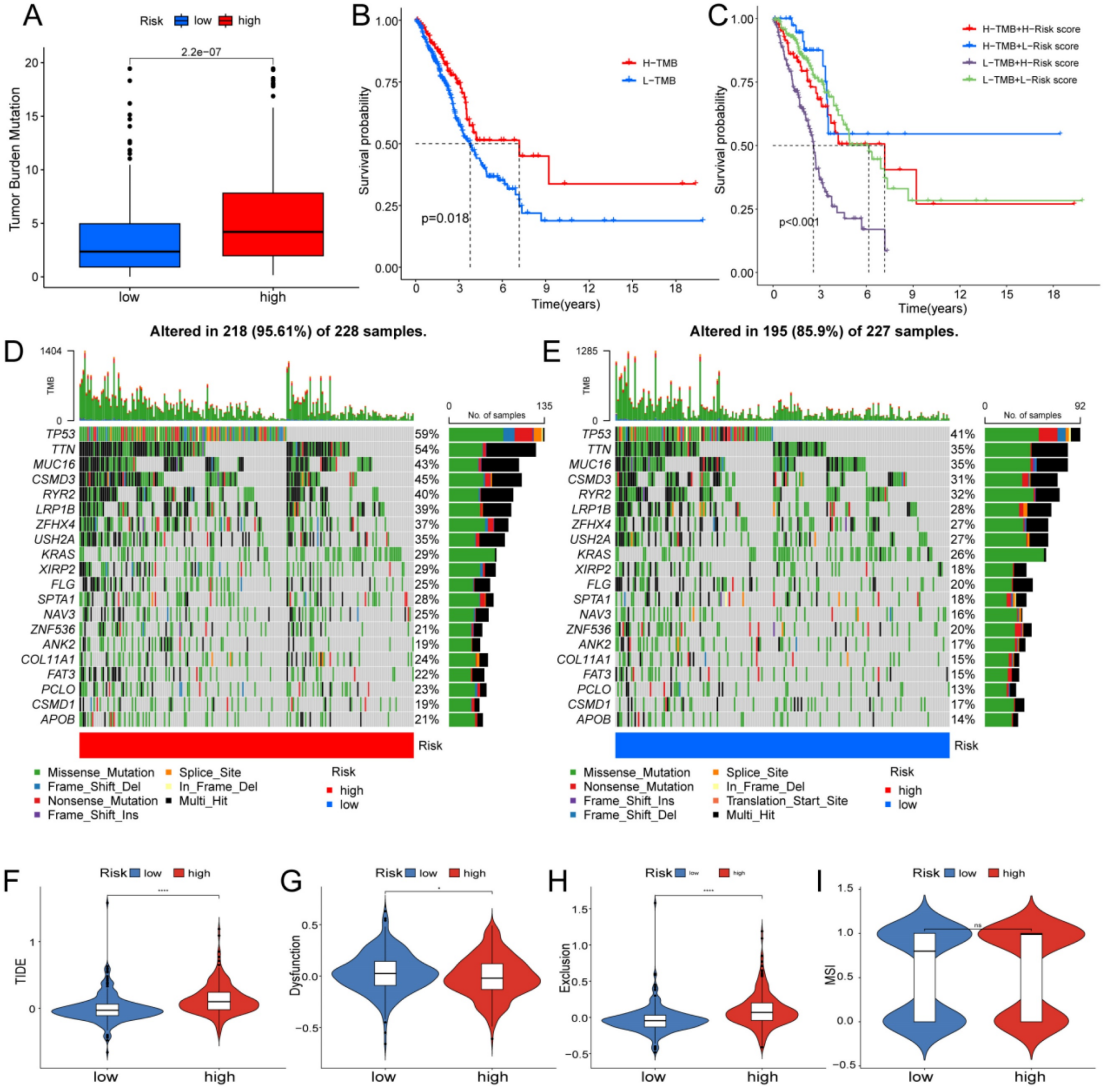
** Determination of the relationship between the hypoxia and glycolysis related prognostic model and immunotherapy. (A)** The TMB difference in the high risk and low risk groups tested by the Wilcoxon rank-sum. **(B)** Kaplan-Meier plots of OS for patients in the high TMB group and low TMB group. **(C)** Kaplan-Meier plots of OS for patients in the risk groups and TMB groups. **(D, E)** The waterfall plot shows the top 20 genes mutated and their difference in the TCGA high risk and low risk groups. (F-I) Boxplots show the differences in the **(F)** TIDE, **(G)** Dysfunction, **(H)** Exclusion, **(I)** MSI between risk groups; ^ns^p ≥ 0.05, *p < 0.05, ****p < 0.0001.

Based on clinical trials and preclinical investigations, immune checkpoint blockade has been shown to provide prolonged clinical benefits, encompassing treatment responses and extended survival, particularly to patients with higher TMB 27, 28. We investigated the difference in TMB between different risk score groups, and subsequent Wilcoxon testing revealed that the group with higher risk scores had a higher TMB (Fig. 7A).

**Figure 8:**
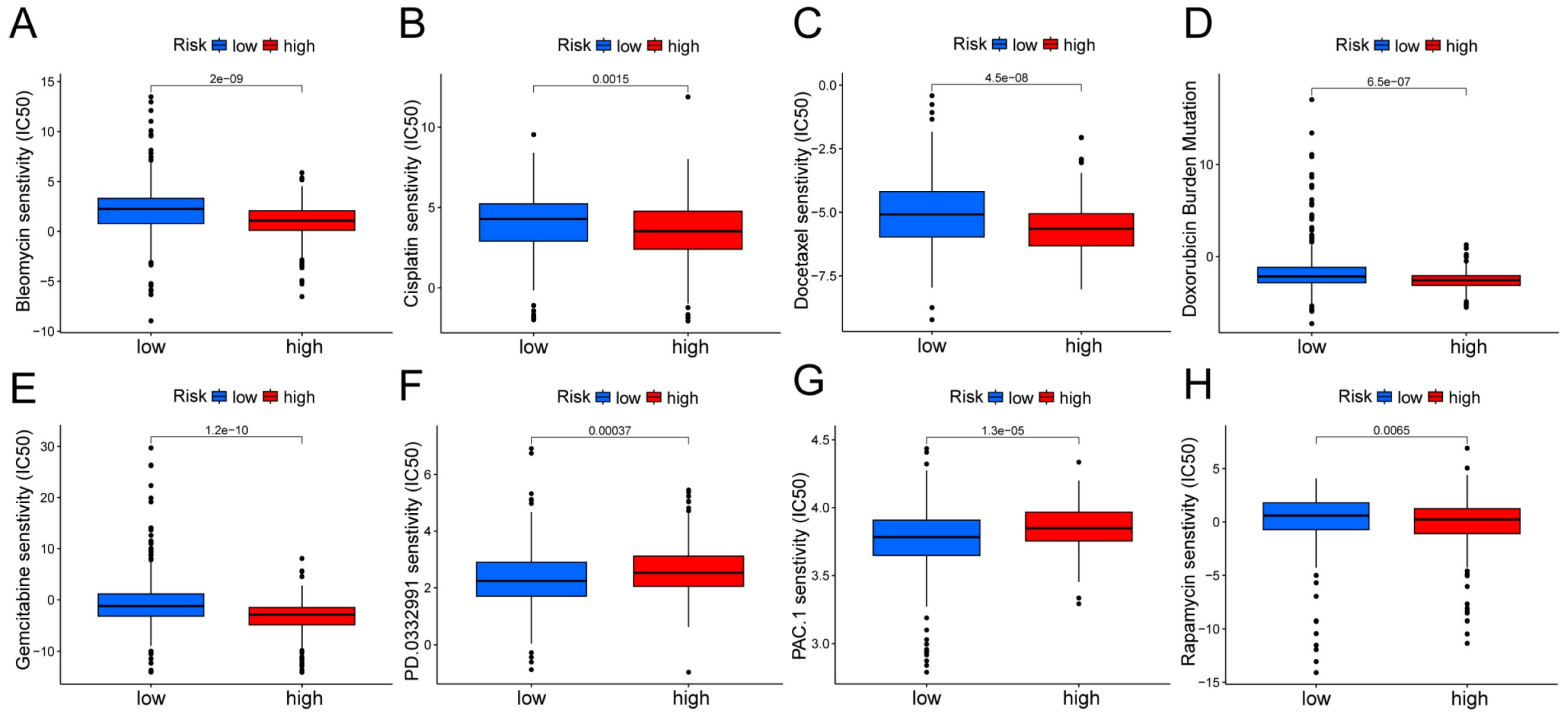
Boxplots show the differences in the estimated IC50 levels of **(A)** bleomycin, **(B)** cisplatin, **(C)** docetaxel, **(D)** doxorubicin, **(E)** gemcitabine, **(F)** PD.0332991, **(G)** PAC.1, and **(H)** rapamycin between risk groups.

In the Kaplan Meier analysis, the survival outcome of the high TMB subgroup was better (Fig. 7B). Combine the TMB and risk score in the Kaplan Meier analysis, the survival outcome of the patients in both the high TMB group and low risk score group was the best (Fig. 7C). Our findings suggest that the LUAD patients with high risk score may potentially exhibit increased responsiveness to immunotherapy. We conducted an exploration of the mutation characteristics within the TCGA-LUAD cohort. The top 20 mutated genes were identified. Notably, TP53 exhibited the highest mutation frequency, accounting for approximately 59% in the high risk score group and 41% in the low risk score group, followed closely by TTN with a mutation rate of about 54% in the high risk score group and 35% in the low risk score group (Fig. 7D, E). Among these alterations, missense mutations were the most prevalent variant classification. Significantly, the distribution of mutations in most of these 20 genes within the high and low risk groups displayed statistically meaningful differences. We employed the TIDE algorithm to evaluate the response to immunotherapy between the subgroups, revealing a statistically significant difference between the two groups (Fig. 7F). Furthermore, we discovered significant differences in the T cell dysfunction, T-cell exclusion score, and MSI between the two risk groups (Fig. 7G-I). The “pRRophetic” R package which described by Geeleher et al. was used to predict drug effects in LUAD patients based on the drug response prediction formula. We found that Bleomycin, Cisplatin, Docetaxel, Doxorubicin, Gemcitabine, and Rapamycin showed high sensitivity in the low risk score group, while PD.0332991, and PAC.1 showed high sensitivity in the high risk score group (Fig. 8A-H).

## Discussion

LUAD is a complex and heterogeneous disease characterized by a multifaceted molecular landscape. The intricate interplay between hypoxia and glycolysis has garnered increasing attention as a key contributor to LUAD progression. In this study, we successfully identified hypoxia and glycolysis related gene signatures and constructed a robust prognostic model. This discussion delves deeper into the implications of our findings, their potential clinical applications, and directions for future research.

Given the complex and multifaceted nature of hypoxia within the tumor microenvironment, relying on a single biomarker for hypoxia assessment is not considered valid 29. To address this issue, we employed the t-SNE algorithm, a classical type of machine learning technique known for its robust dimensionality reduction capabilities. The t-SNE algorithm has previously proven its utility in subtype classification across various cancer types, including prostate cancer, breast cancer, and gastric cancer 30, 31. To identify the hypoxia status, the K-Means Clustering (K-means) algorithm was employed. We chose the WSS (Within Sum of Squares) method to determine the number of clusters. When K>2, the downward trend of WSS becomes more gradual. thus, adding a cluster for “K=2” might not be meaningful. In the end, we selected “K=2” as the final result. As for the glycolysis status, we utilized the K-means machine learning algorithm to conduct an unsupervised consensus clustering. This method enables the categorization and condensation of cases into distinct clusters based on provided hallmarks or signatures, and it has found widespread application in cancer research, including melanoma and pancreatic adenocarcinoma 32, 33. Regarding the choice of “K=2”, we employed a comprehensive evaluation, considering the Item-Consensus plot, PAC algorithm, and the relative change in the area under the cumulative distribution function curves. The decision to divide the samples into two groups was based on the optimal clustering results obtained through this iterative process. Through these methods, we successfully categorized LUAD patients into distinct groups based on their hypoxia and glycolysis status. The Kaplan-Meier plots demonstrates that our clustering can be effectively distinguished. Furthermore, our approach holds promise as a valuable reference for follow-up studies.

This study has identified key signature genes in LUAD that exhibit associations with hypoxia and glycolysis. These genes are GAPDH, FSCN1, SLC2A1, FAM83A, PLEK2, and GJB3, some of which have been previously documented in various cancer types. Fascin Actin-bundling Protein 1 (FSCN1), an actin bundling protein, plays a pivotal role in cell-cell interactions, adhesion, and motility by regulating the function of filopodial protrusions and microfilaments 34. These cellular processes are directly implicated in the invasion and metastasis of various cancer types. Consequently, FSCN1 has emerged as a promising candidate for both prognostic assessment and therapeutic intervention in patients with various tumors. In numerous malignant tumors, FSCN1 exhibits upregulation, suggesting its potential as an oncogenic factor, as it fosters tumor cell migration and invasion. Solute carrier family 2 member 1 (SLC2A1), commonly referred to as glucose transporter protein type 1, has been consistently associated with the occurrence and prognosis of pancreatic cancer in numerous studies 35-37. Family with sequence similarity 83, member A (FAM83A), also known as BJ-TSA-9, is situated on chromosome 8q24. Initially, it garnered attention as a candidate tumor-specific gene through a bioinformatics approach. Moreover, FAM83A exhibits notable overexpression in various human tumors, such as lung, breast, testis, and bladder cancer 38-40, suggesting that FAM83A potentially plays an oncogenic role in the initiation and advancement of cancer. Pleckstrin-2 (PLEK2) belongs to the pleckstrin family, characterized by the presence of pleckstrin homology (PH) and disheveled-Egl-10-pleckstrin (DEP) domains. Current research predominantly centers on aspects related to cell motility, encompassing the dynamics, migration, and metastasis of both red blood cells and tumors. PLEK2 has been documented in the context of colorectal cancer, esophageal cancer, and gallbladder cancer 41-43.

We developed a prognostic risk model using these six genes. The TCGA cohort revealed 1-, 3-, and 5-year OS rates of 0.700, 0.719, and 0.668, respectively. In validation cohort GSE31210, the area under the curve (AUC) values for 1-, 3-, and 5-year OS were 0.815, 0.682, and 0.739. Moreover, compared with other similar research, our hypoxia and glycolysis related prognostic model had better effectiveness. Liu et al. developed a prognostic model based on hypoxia-associated genes, and the AUC values of this model were 0.66, 0.72, and 0.62, respectively, for 1-, 3-, and 5-year OS 14. Zhu et al. constructed a prognostic risk model based on glycolysis-related genes, and the AUC values of this model were 0.60, 0.64, and 0.76, respectively, for 1-, 3-, and 5-year OS 44. Subsequently, we incorporated our prognostic risk model and relevant clinical features to create a nomogram. The calibration of the nomogram demonstrates excellent prediction of 1-year and 5-year survival rates for LUAD patients in TCGA cohort. However, the prediction accuracy for the 3-year survival rate is slightly lower. This could be attributed to the relatively fewer occurrences of patient deaths within the shorter time frame, potentially limiting the model's predictive accuracy within the 3-year period. As time progresses, the increase in both sample size and events may enhance the predictive accuracy for the 5-year survival rate. Additionally, patients may undergo various changes between the 3-year and 5-year marks, such as alterations in treatment plans and evolution of disease status. These changes might introduce more uncertainty when predicting future disease progression, affecting the model's accuracy. As for the GSE31210 cohort, our model effectively predicts 1-year and 3-year survival rates, but exhibits suboptimal performance in predicting the 5-year survival rate. The model may excel in predicting shorter-term survival rates but encounter increased uncertainty when forecasting over longer durations. Long-term predictions are susceptible to various factors, including treatment advancements and fluctuations in patient conditions, which could contribute to a decline in accuracy. In summary, the model we constructed demonstrates robust prognostic prediction for LUAD patients, with the understanding that certain limitations may exist, particularly in the context of predicting intermediate-term survival rates. These findings collectively affirm the successful development of a prognostic risk model associated with hypoxia and glycolysis. Immunotherapy has gained increasing prominence as a treatment modality for advanced LUAD. Nevertheless, a considerable portion of patients does not derive benefits from PD-1/PD-L1 immune checkpoint inhibitors due to the limited universality of immunotherapy. This underscores the imperative for identifying an alternative costimulatory signal within the TME, which requires urgent investigation 45. In our research, we conducted an analysis of the correlations between immune checkpoints and the risk model, leading to the identification of two subtypes with markedly distinct expressions of immune checkpoints, including CD276, TNFRSF14, CD274, BTLA, IDO2 and so on. Next, we found that our risk score was associated with TMB, suggesting that our signature appeared to guide immunotherapy. The TIDE analysis was used to confirm the above points of our view. These evidences affirmed that our risk score could bring hope to precisely targeted therapy.

Nonetheless, this study does have certain limitations. While the utilization of multiomics profiling, distinct expression profiles, and advanced bioinformatic methods has greatly enhanced the development of novel prognostic models for LUAD patients 46, it is essential to acknowledge that most studies to date have relied on comprehensive genomic and transcriptional datasets obtained from various databases, often lacking detailed insights into the underlying biological processes. Consequently, the resulting signatures may inherently exhibit some bias, as they do not account for the intrinsic characteristics of cancer. It is also worth noting that all samples included in this study are based on retrospective data. Therefore, there is a clear imperative for large-scale experimental studies to validate the findings presented herein.

In conclusion, a robust and validated scoring system has been developed to predict OS in LUAD. This score holds the potential to serve as a dependable biomarker for predicting patient survival, aiding in the formulation of highly personalized treatment plans. Furthermore, it contributes to a deeper comprehension of immune infiltrations within the TME and may offer valuable insights for the exploration of more effective immunotherapeutic strategies.

## Conclusions

In conclusion, our study advances our understanding of LUAD by highlighting the critical role of hypoxia and glycolysis related genes and providing a practical prognostic model for clinical use. This model has the potential to change risk stratification and treatment decision-making in LUAD, ultimately improving patient outcomes. Continued research efforts in this direction may pave the way for more effective therapeutic strategies and contribute to the ongoing battle against LUAD.

## Supplementary Material

Supplementary figures and tables.

## Funding

This work was supported by the National Natural Science Foundation of China (82172569, 82072595, and 61973232), Tianjin Key Medical Discipline (Specialty) Construction Project, and Tianjin Health Science and Technology Project (ZC20179).

## Data Availability Statement

Publicly available datasets were analyzed in this study. This data can be found here: https://portal.gdc.cancer.gov/.

## Author Contributions

This project was conceived by JC and HL. WZ, CD, and MZ wrote the manuscript. YL, HH, XL, and ZS collected and analyzed data. WG and QC revised the manuscript. All authors have read and agreed to the published version of the manuscript.

## Figures and Tables

**Table 1:** The association of clinicopathological features in TCGA cohort.

Characteristics	TCGA-LUAD cohort		
	High risk (%)	Low risk (%)	P value
**Age**			
≤65 year	131 (53.7%)	112 (45.7%)	0.0778
>65 year	113 (46.3%)	133 (54.3%)	
**Gender (%)**			
Female	119 (48.8%)	143 (58.4%)	0.0334
Male	125 (51.2%)	102 (41.6%)	
**Pathologic stage**			
Ⅰ	111 (45.5%)	161 (65.7%)	<0.0001
Ⅱ	71 (29.1%)	46 (18.8%)	
Ⅲ	47 (19.3%)	28 (11.4%)	
Ⅳ	15 (6.1%)	10 (4.1%)	
**T stage**			0.0002
T1	61 (25.0%)	107 (43.7%)	
T2	143 (58.6%)	114 (46.5%)	
T3	31 (12.7%)	14 (5.7%)	
T4	8 (3.3%)	8 (3.3%)	
TX	1 (0.4%)	2 (0.8%)	
**N stage**			0.0015
N0	143 (58.6%)	180 (73.5%)	
N1	56 (23.0%)	33 (13.5%)	
N2	41 (16.8%)	23 (9.4%)	
N3	1 (0.4%)	1 (0.4%)	
Nx	3 (1.2%)	8 (3.3%)	
**M stage**			0.4295
M0	162 (66.4%)	164 (66.9%)	
M1	15 (6.1%)	9 (3.7%)	
Mx	67 (27.5%)	72 (29.4%)	
			

**Table 2:** The association of clinicopathological features in GSE31210 cohort.

Characteristics	GSE31210 cohort		
	High risk (%)	Low risk (%)	P value
**Age**			
≤65 year	87 (77.0%)	89 (78.8%)	0.7486
>65 year	26 (23.0%)	24 (21.2%)	
**Gender (%)**			
Female	53 (46.9%)	68 (60.2%)	0.0454
Male	60 (53.1%)	45 (39.8%)	
**Pathologic stage**			
Ⅰ	63 (55.8%)	105 (92.9%)	<0.0001
Ⅱ	50 (44.2%)	8 (7.1%)	
