# Supplementary material for: Identification and Validation of a Hypoxia and Glycolysis Prognostic Signatures in Lung Adenocarcinoma
Source: J Cancer. 2024 Jan 21;15(6):1568–82. doi: 10.7150/jca.91504 (PMC10869968; doi:10.7150/jca.91504)
Supplement: Supplementary file 1 — Supplementary figures and tables. [file jcav15p1568s1.pdf]

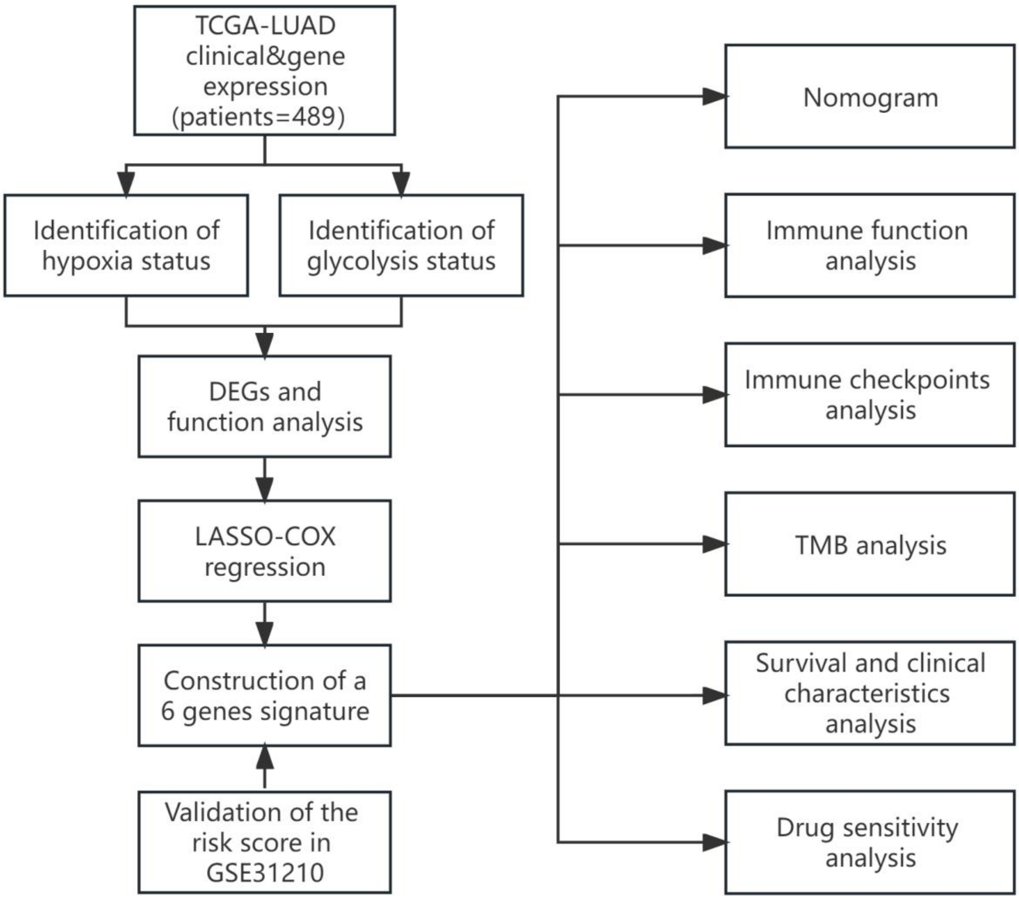

Fig. S1

The entire analytical process of the study.

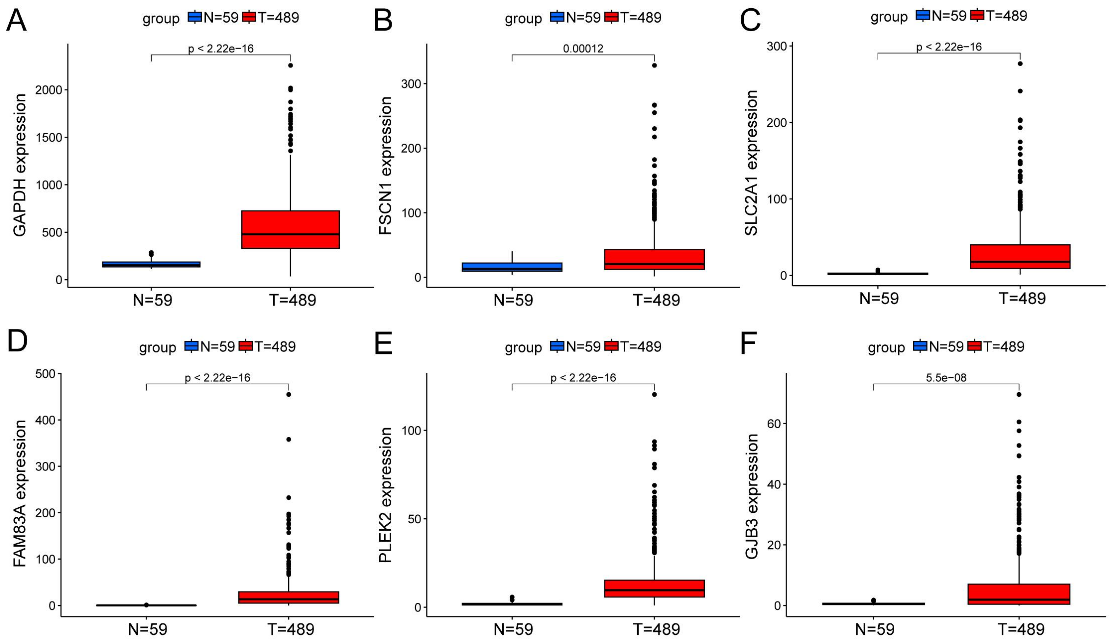

Fig. S2

The expression levels of (A) GAPDH, (B) FSCN1, (C) SLC2A1, (D) FAM83A, (E) PLEK2, and (F) GJB3 in tumor tissues and normal tissues.

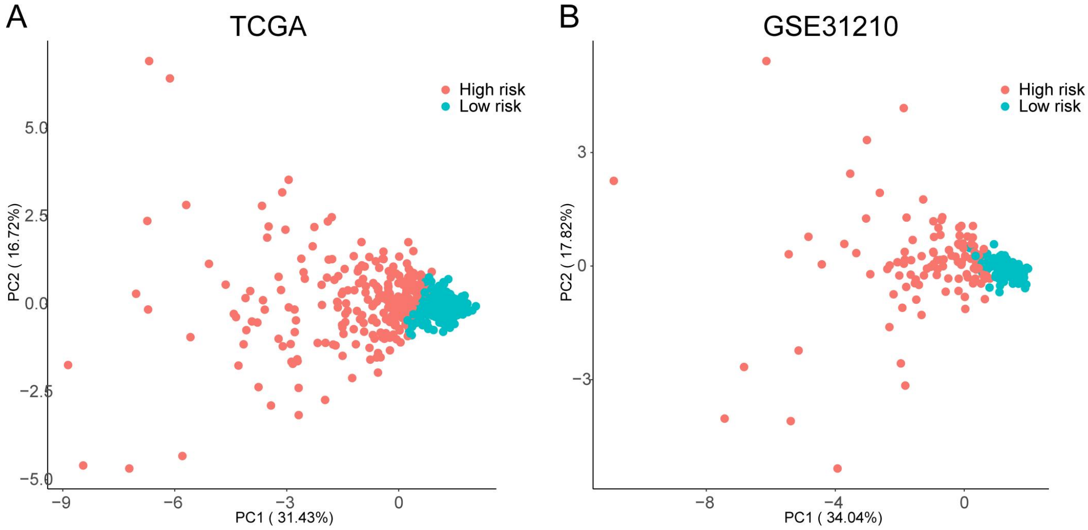

Fig. S3

PCA analysis demonstrating significant differences between high risk and low risk patients in the (A) TCGA cohort and (B) GSE31210 cohort.

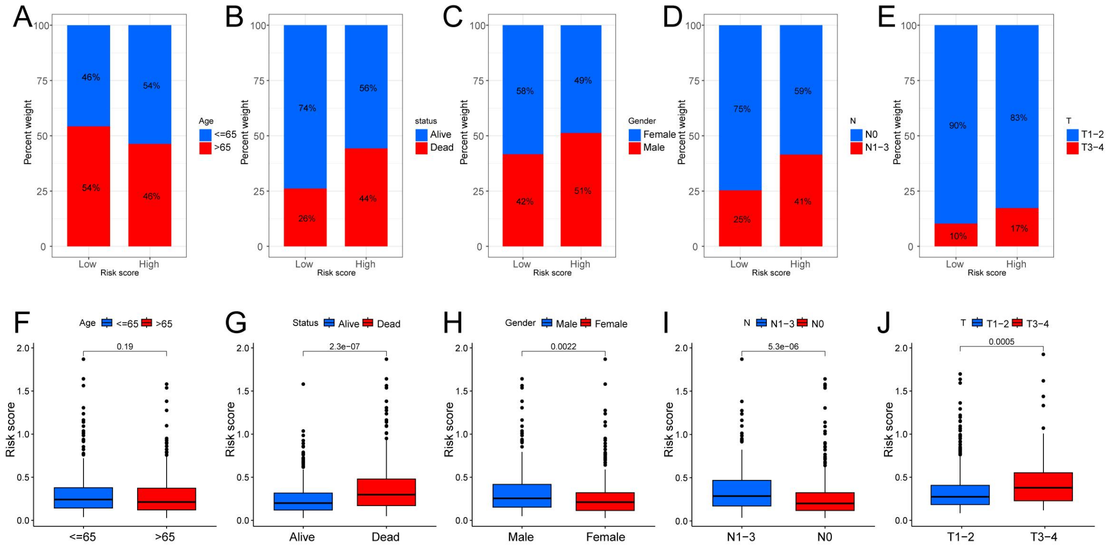

Fig. S4

Clinical subgroup analysis encompassing clinicopathological characteristics. (A-E) The percentage of clinicopathological characteristics in low risk and high risk groups. (F-J) Boxplots show the differences in the risk score between clinical subgroups.

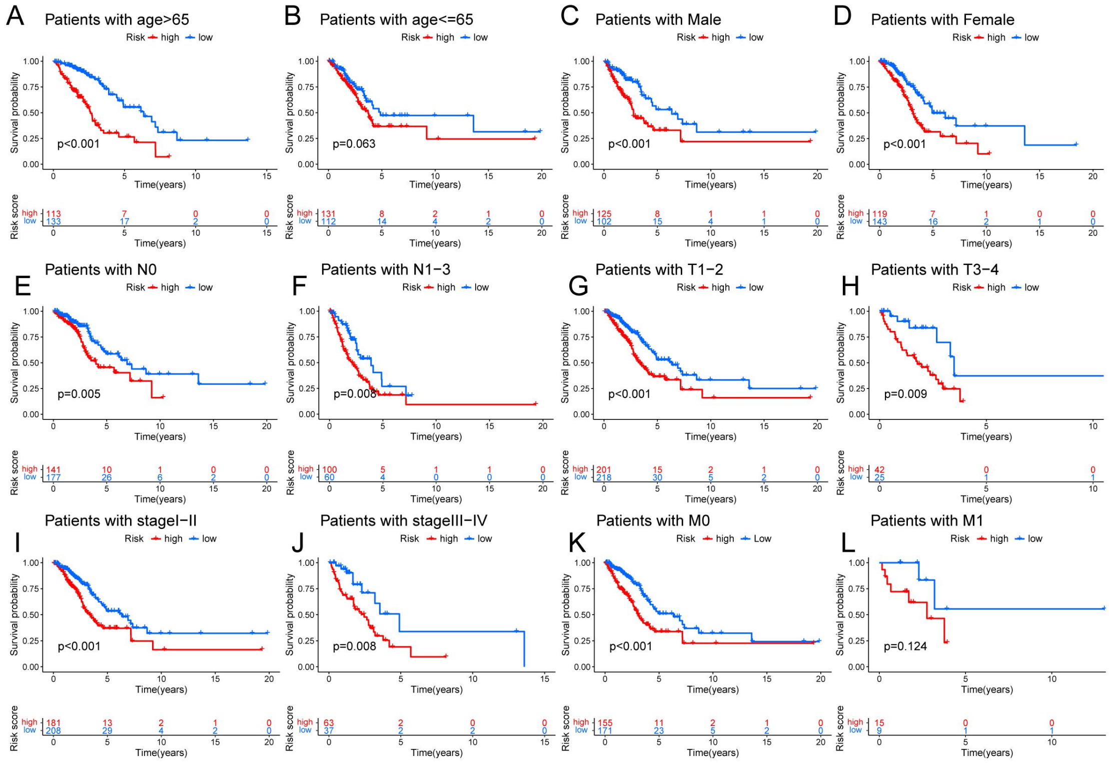

Fig. S5

Kaplan–Meier plots of overall survival for patients in the high risk and low risk groups within (A, B) age, (C, D) gender, (E, F) N stage, (G, H) T stage, (I, J) clinical stage, (K, L) M stage subgroups.

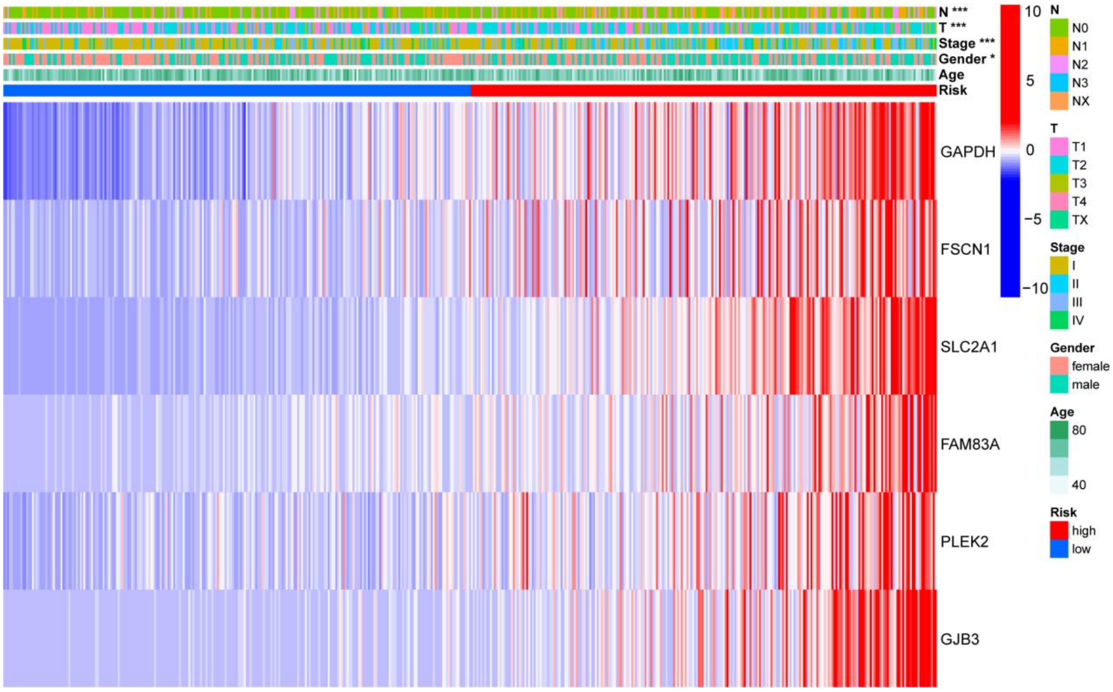

Fig. S6

The heatmap of differences in clinical characteristics and the hypoxia and glycolysis related genes expression between the high risk and low risk groups. *$p < 0.05$, ***$p < 0.001$.

Table S1. Summary of hypoxia-related genes.

Hypoxia-related genes				
ADM	DCN	HK1	NDST2	SAP30
ADORA2B	DDIT3	HK2	NEDD4L	SCARB1
AK4	DDIT4	HMOX1	NFIL3	SDC2
AKAP12	DPYSL4	HOXB9	NR3C1	SDC3
ALDOA	DTNA	HS3ST1	P4HA1	SDC4
ALDOB	DUSP1	HSPA5	P4HA2	SELENBP1
ALDOC	EDN2	IDS	PAM	SERPINE1
AMPD3	EFNA1	IER3	PCK1	SIAH2
ANGPTL4	EFNA3	IGFBP1	PDGFB	SLC25A1
ANKZF1	EGFR	IGFBP3	PDK1	SLC2A1
ANXA2	ENO1	IL6	PDK3	SLC2A3
ATF3	ENO2	ILVBL	PFKFB3	SLC2A5
ATP7A	ENO3	INHA	PFKL	SLC37A4
B3GALT6	ERO1A	IRS2	PFKP	SLC6A6
B4GALNT2	ERRFI1	ISG20	PGAM2	SRPX

BCAN	ETS1	JMJD6	PGF	STBD1
BCL2	EXT1	JUN	PGK1	STC1
BGN	F3	KDELR3	PGM1	STC2
BHLHE40	FAM162A	KDM3A	PGM2	SULT2B1
BNIP3L	FBP1	KIF5A	PHKG1	TES
BRS3	FOS	KLF6	PIM1	TGFB3
BTG1	FOSL2	KLF7	PKLR	TGFBI
CA12	FOXO3	KLHL24	PKP1	TGM2
CASP6	GAA	LALBA	PLAC8	TIPARP
CAV1	GALK1	LARGE1	PLAUR	TKTL1
CCNG2	GAPDH	LDHA	PLIN2	TMEM45A
NOCT	GAPDHS	LDHC	PNRC1	TNFAIP3
CDKN1A	GBE1	LOX	PPARGC1A	TPBG
CDKN1B	GCK	LXN	PPFIA4	TPD52
CDKN1C	GCNT2	MAFF	PPP1R15A	TPI1
CHST2	GLRX	MAP3K1	PPP1R3C	TPST2
CHST3	GPC1	MIF	PRDX5	UGP2
CITED2	GPC3	MT1E	PRKCA	VEGFA
COL5A1	GPC4	MT2A	CAVIN3	VHL
CP	GPI	MXI1	CAVIN1	VLDLR
CSRP2	GRHPR	MYH9	PYGM	CCN5
CCN2	GYS1	NAGK	RBPJ	WSB1
CXCR4	HAS1	NCAN	RORA	XPNPEP1
ACKR3	HDLBP	NDRG1	RRAGD	ZFP36
CCN1	HEXA	NDST1	S100A4	ZNF292

Table S2. Summary of glycolysis-related genes.

Glycolysis-related genes				
ABCB6	EGLN3	ME2	SRD5A3	RANBP2
ADORA2B	ELF3	MED24	STC1	NUP205
AGL	ENO1	MERTK	STC2	HK1
AGRN	ENO2	MET	STMN1	SEC13
AK3	ERO1A	MIF	TALDO1	ADPGK
AK4	EXT1	MIOX	TFF3	HK3
AKR1A1	EXT2	MPI	TGFA	NUP35
ALDH7A1	FAM162A	MXI1	TGFBI	GNPDA2
ALDH9A1	FBP2	NANP	TKTL1	PRKACG
ALDOA	FKBP4	NASP	TPBG	PGM2L1
ALDOB	FUT8	NDST3	TPI1	PFKFB3
ALG1	G6PD	NDUFV3	TPST1	PGK2
ANG	GAL3ST1	NOL3	GFUS	BPGM
ANGPTL4	GALE	NSDHL	TXN	PGP

ANKZF1	GALK1	NT5E	UGP2	POM121
ARPP19	GALK2	P4HA1	VCAN	NUP62
ARTN	GAPDHS	P4HA2	VEGFA	POM121C
AURKA	GCLC	PAM	VLDLR	ADH1A
B3GALT6	GFPT1	PAXIP1	XYLT2	ADH1B
B3GAT1	GLCE	PC	ZNF292	ADH1C
B3GAT3	GLRX	PDK3	NUP160	ADH4
B3GNT3	GMPPA	PFKFB1	TPR	ADH5
B4GALT1	GMPPB	PFKP	NDC1	ADH6
B4GALT2	GNE	PGAM1	NUP133	GALM
B4GALT4	GNPDA1	PGAM2	PRKACA	ADH7
B4GALT7	GOT1	PGK1	NUP37	LDHAL6A
BIK	GOT2	PGLS	GCKR	DLAT
BPNT1	GPC1	PGM2	SEH1L	ALDH2
CACNA1H	GPC3	PHKA2	NUP50	ALDH3A1
CAPN5	GPC4	PKM	AAAS	ALDH1B1
CASP6	GPR87	PKP2	NUP188	ALDH1A3
CD44	GUSB	PLOD1	RAE1	FBP1
CDK1	GYS1	PLOD2	NUP93	ALDH3B1
CENPA	GYS2	PMM2	GPI	ALDH3B2
CHPF	HAX1	POLR3K	PPP2R1A	ALDH3A2
CHPF2	HDLBP	PPFIA4	GCK	G6PC1
CHST1	HK2	PPIA	ENO3	LDHB
CHST12	HMMR	PPP2CB	NUP88	PGAM4
CHST2	HOMER1	PRPS1	ALDOC	PCK1
CHST4	HS2ST1	PSMC4	NUP98	PCK2
CHST6	HS6ST2	PYGB	NUP107	PDHA1
CITED2	HSPA5	PYGL	GAPDH	PDHA2
CLDN3	IDH1	QSOX1	PPP2R5D	PDHB
CLDN9	IDUA	RARS1	NUP155	PGM1
CLN6	IER3	RBCK1	PPP2CA	ACSS2
COG2	IGFBP3	RPE	PFKFB4	G6PC2
COL5A1	IL13RA1	RRAGD	NUP43	ACSS1
COPB2	IRS2	SAP30	PFKFB2	LDHAL6B
CTH	ISG20	SDC1	NUP153	MPC2
CXCR4	KDELR3	SDC2	NUP85	MPC1
CYB5A	KIF20A	SDC3	NUP214	SLC2A1
DCN	KIF2A	SDHC	NUP210	SLC2A2
DDIT4	LCT	SLC16A3	NUP42	SLC2A3
DEPDC1	LDHA	SLC25A10	PPP2R1B	SLC2A4
DLD	LDHC	SLC25A13	NUP54	SLC2A5
DPYSL4	LHPP	SLC35A3	NUP58	
DSC2	LHX9	SLC37A4	PFKL	

ECD	MDH1	SOD1	PRKACB	
EFNA3	MDH2	SOX9	PKLR	
EGFR	ME1	SPAG4	PFKM	

Table S3. Genes identified through univariate Cox regression analysis.

gene	HR	L95CI	H95CI	pvalue
SFTPA1	0.999885653	0.999776022	0.999995296	0.040949145
SFTPC	0.99961211	0.999296353	0.999927967	0.016090465
GAPDH	1.000940659	1.000610798	1.00127063	2.26E-08
TPI1	1.002459067	1.001324854	1.003594565	2.12E-05
AGER	0.996380467	0.993176817	0.99959445	0.027325943
SCGB1A1	0.998075396	0.996391673	0.999761965	0.025330907
HMGA1	1.001919749	1.001020757	1.002819548	2.82E-05
BTG2	0.994082089	0.990306745	0.997871825	0.002233068
SELENBP1	0.996165711	0.993239401	0.999100642	0.010485056
MFAP4	0.995334432	0.991366053	0.999318697	0.021771427
TXNRD1	1.002119973	1.001024464	1.00321668	0.000147783
SUSD2	0.995136679	0.991405004	0.998882401	0.010980186
CYP4B1	0.996151059	0.992942151	0.999370338	0.019151082
CLIC6	0.995139639	0.990991828	0.999304811	0.022236905
TK1	1.008664422	1.004280193	1.013067792	0.000103733
FSCN1	1.009126626	1.006065242	1.012197325	4.61E-09
UBE2C	1.004135029	1.000531702	1.007751334	0.02446348
SLC7A5	1.007297833	1.003994925	1.010611607	1.43E-05
SLC2A1	1.011298709	1.008000757	1.014607451	1.57E-11
NDNF	0.991080353	0.983733285	0.998482294	0.018272826
AQP4	0.990790714	0.98179499	0.999868861	0.046795518
PFN2	1.006074828	1.000962286	1.011213482	0.019806727
CYP24A1	1.002761098	1.001193241	1.00433141	0.00055299
ANGPTL4	1.007164758	1.003811676	1.010529041	2.72E-05
ASPH	1.007551176	1.002926686	1.012196989	0.00135046
FAM83A	1.009732052	1.006949024	1.012522772	6.09E-12
CDA	1.003989846	1.001017645	1.006970873	0.008479181
CDH3	1.005207548	1.000436778	1.010001068	0.032365164
RHOV	1.004346899	1.002475322	1.00622197	5.17E-06
PFKP	1.014999283	1.007611225	1.022441511	6.49E-05
C7	0.986955993	0.976546201	0.997476751	0.015225985
FKBP4	1.012457026	1.006345557	1.018605609	6.13E-05
PTGR1	1.00440393	1.000989286	1.007830222	0.011437124
CDC20	1.00997531	1.003549192	1.016442576	0.002304821
PLOD2	1.006149885	1.00164509	1.01067494	0.007408217
CCNB1	1.016115927	1.008160269	1.024134365	6.71E-05

TPX2	1.010673915	1.004866394	1.016515	0.000304957
NFIX	0.983930292	0.968834381	0.999261421	0.040012026
GGTLC1	0.985406035	0.974397663	0.996538774	0.010321539
MFSD2A	0.985228941	0.970766615	0.999906724	0.04857227
CDCP1	1.026167564	1.014808513	1.037653761	5.41E-06
FHL1	0.967612136	0.94171771	0.994218581	0.017363872
PLEK2	1.0225513	1.014804321	1.03035742	9.06E-09
CACNA2D2	0.979142362	0.963950654	0.994573488	0.008242161
GCLC	1.007236103	1.002053158	1.012445855	0.006159191
LRRK2	0.984685572	0.970973124	0.998591672	0.031010854
BIRC5	1.019303524	1.007147905	1.031605854	0.001786758
TNNT1	1.011597908	1.005125025	1.018112476	0.000430286
CLEC3B	0.928002513	0.888249527	0.969534616	0.00082281
PMAIP1	1.012482246	1.001595871	1.023486945	0.02450799
EGLN3	1.006609523	1.001263863	1.011983723	0.015313296
FMO2	0.944516095	0.908530763	0.981926744	0.003973803
LOXL2	1.019997829	1.013559634	1.02647692	8.85E-10
ATP13A4	0.974597785	0.95678334	0.992743919	0.006263006
DLC1	0.967419789	0.941288905	0.994276085	0.017747495
ADH1B	0.968081219	0.939411885	0.997625495	0.034433372
SFTA1P	0.979752146	0.960895198	0.998979149	0.039115454
ANLN	1.035086865	1.022050731	1.048289274	9.67E-08
LYPD3	1.010687099	1.005070335	1.016335252	0.000184988
CHRDL1	0.970949923	0.945697585	0.996876557	0.02833353
TNS4	1.016930453	1.010883025	1.02301406	3.45E-08
MTURN	0.945052077	0.909302028	0.982207673	0.004073585
CCNA2	1.035306388	1.018349013	1.052546135	3.82E-05
FOXM1	1.03348165	1.016604235	1.050639259	8.85E-05
RRM2	1.040447203	1.01979885	1.061513632	0.000105786
KIF2C	1.021870223	1.003707408	1.040361707	0.018059802
CGNL1	0.920119779	0.871528734	0.971419959	0.00263441
CDCA5	1.04028728	1.019876122	1.061106933	9.36E-05
AHNAK2	1.040718427	1.025786774	1.055867428	6.20E-08
PLEKHS1	0.978618938	0.958147856	0.99952739	0.045092432
GJB3	1.035007101	1.024303723	1.045822322	8.73E-11
CDKN3	1.042067953	1.019386666	1.065253898	0.000242445
SMOX	1.030527545	1.017578342	1.043641534	3.15E-06
ARNTL2	1.038316353	1.02337781	1.053472958	3.67E-07
DLGAP5	1.042532551	1.021824359	1.063660413	4.72E-05
ZNF750	0.956289493	0.92685169	0.986662271	0.005084064
PBK	1.037357767	1.017012319	1.058110229	0.000284331
PLK1	1.063181546	1.035178821	1.091941775	6.84E-06
KIF4A	1.06203249	1.030598392	1.094425355	8.63E-05

CDC6	1.024856758	1.002339946	1.047879393	0.030298701
TPPP	0.929993249	0.884387829	0.977950414	0.00466838
MAMDC2	0.930624952	0.876065268	0.988582509	0.019674789
GINS2	1.043128771	1.013664807	1.073449156	0.003872362
VIPR1	0.856412913	0.772752544	0.949130589	0.003122352
MAD2L1	1.05809493	1.02272925	1.094683545	0.001131028
TBX4	0.922804968	0.859300466	0.991002616	0.027215371
HMMR	1.080959991	1.045950971	1.117140798	3.58E-06
ITGA8	0.893951543	0.829424909	0.963498144	0.003359662
MS4A1	0.958221112	0.924457184	0.9932182	0.01971279
SELP	0.92333206	0.855694166	0.996316355	0.039874953
PRR11	1.09231996	1.043191647	1.143761933	0.000169307
SPATA18	0.946180784	0.898700792	0.996169231	0.035197366
CYS1	1.02694416	1.005236803	1.049120272	0.014722556
PKP2	1.050130688	1.027643252	1.073110206	9.47E-06
COL4A3	0.914739333	0.857049906	0.976311929	0.007334847
NCAPG	1.049685824	1.013784929	1.086858068	0.006313537
SCN7A	0.88601332	0.811635834	0.967206683	0.006824276
COL7A1	1.026667287	1.005200585	1.048592425	0.014643069
ALDOA	1.085673281	1.043794967	1.129231803	4.21E-05
ORC1	1.067984869	1.019277862	1.119019379	0.005750145
CFTR	0.92932848	0.875532363	0.986430041	0.015994346
FAM189A2	0.784836473	0.680913686	0.904620221	0.000828343
EXO1	1.061125299	1.026068238	1.097380133	0.000537586
CD302	0.829974124	0.745466464	0.924061751	0.000670345
EFCC1	0.794839686	0.684596711	0.922835468	0.00257743
RAD51	1.101917115	1.041411386	1.165938211	0.000756622
CDCA3	1.091872571	1.032430742	1.154736742	0.002087889
SKA1	1.071951497	1.023884193	1.12227537	0.002994085
DAPK2	0.833612097	0.727393743	0.955341085	0.008872465
FAM184A	0.827487451	0.738244187	0.927518962	0.001145061
PGM5	0.86779792	0.773217492	0.973947482	0.016026389
CX3CR1	0.833832402	0.740962033	0.938342915	0.002559058
EP300-AS1	0.871878685	0.7673283	0.99067432	0.035402192
MTFR2	1.101872116	1.020762833	1.1894263	0.012891875

Table S4. Composition and correlation coefficients of the model genes.

Gene	Coef
GAPDH	0.00012644
FSCN1	0.000598786
SLC2A1	0.00254632
FAM83A	0.002353383
PLEK2	0.001549281

GJB3	0.009291976
